# Radiomics analysis of contrast-enhanced CT scans can distinguish between clear cell and non-clear cell renal cell carcinoma in different imaging protocols

**DOI:** 10.3389/fmed.2022.974485

**Published:** 2022-10-13

**Authors:** Bettina Katalin Budai, Róbert Stollmayer, Aladár Dávid Rónaszéki, Borbála Körmendy, Zita Zsombor, Lõrinc Palotás, Bence Fejér, Attila Szendrõi, Eszter Székely, Pál Maurovich-Horvat, Pál Novák Kaposi

**Affiliations:** ^1^Department of Radiology, Faculty of Medicine, Medical Imaging Centre, Semmelweis University, Budapest, Hungary; ^2^Department of Urology, Faculty of Medicine, Semmelweis University, Budapest, Hungary; ^3^Department of Pathology, Forensic and Insurance Medicine, Faculty of Medicine, Semmelweis University, Budapest, Hungary

**Keywords:** renal cell carcinoma, computed tomography, radiomics analysis, texture analysis, machine learning, artificial intelligence

## Abstract

**Introduction:**

This study aimed to construct a radiomics-based machine learning (ML) model for differentiation between non-clear cell and clear cell renal cell carcinomas (ccRCC) that is robust against institutional imaging protocols and scanners.

**Materials and methods:**

Preoperative unenhanced (UN), corticomedullary (CM), and excretory (EX) phase CT scans from 209 patients diagnosed with RCCs were retrospectively collected. After the three-dimensional segmentation, 107 radiomics features (RFs) were extracted from the tumor volumes in each contrast phase. For the ML analysis, the cases were randomly split into training and test sets with a 3:1 ratio. Highly correlated RFs were filtered out based on Pearson’s correlation coefficient (*r* > 0.95). Intraclass correlation coefficient analysis was used to select RFs with excellent reproducibility (ICC ≥ 0.90). The most predictive RFs were selected by the least absolute shrinkage and selection operator (LASSO). A support vector machine algorithm-based binary classifier (SVC) was constructed to predict tumor types and its performance was evaluated based-on receiver operating characteristic curve (ROC) analysis. The “Kidney Tumor Segmentation 2019” (KiTS19) publicly available dataset was used during external validation of the model. The performance of the SVC was also compared with an expert radiologist’s.

**Results:**

The training set consisted of 121 ccRCCs and 38 non-ccRCCs, while the independent internal test set contained 40 ccRCCs and 13 non-ccRCCs. For external validation, 50 ccRCCs and 23 non-ccRCCs were identified from the KiTS19 dataset with the available UN, CM, and EX phase CTs. After filtering out the highly correlated and poorly reproducible features, the LASSO algorithm selected 10 CM phase RFs that were then used for model construction. During external validation, the SVC achieved an area under the ROC curve (AUC) value, accuracy, sensitivity, and specificity of 0.83, 0.78, 0.80, and 0.74, respectively. UN and/or EX phase RFs did not further increase the model’s performance. Meanwhile, in the same comparison, the expert radiologist achieved similar performance with an AUC of 0.77, an accuracy of 0.79, a sensitivity of 0.84, and a specificity of 0.69.

**Conclusion:**

Radiomics analysis of CM phase CT scans combined with ML can achieve comparable performance with an expert radiologist in differentiating ccRCCs from non-ccRCCs.

## Introduction

Kidney cancers are one of the most common malignancies in the world accounting for approximately 2.2% of annual cancer diagnoses (431 thousand/year) and 1.8% of cancer-related mortality (179 thousand/year) worldwide. It is almost twice as common in males than in females making it the 11th highest incidence of cancer in men and the 16th in women (1).

Due to the increasing accessibility of non-invasive diagnostic procedures nowadays up to 50% of renal neoplasms are incidentally discovered (2). Many of these small renal masses are benign, but because of their size, they are hard to characterize using imaging modalities increasing the importance of biopsy to select low-risk patients for active surveillance (3). At the time of diagnosis, approximately 15% of patients already have distant metastases (4). Accurate preoperative staging is crucial for making an appropriate treatment decision. For accurate staging – including the assessment of local invasiveness, lymph node involvement, and presence/absence of distant metastases –, contrast-enhanced thoraco-abdominopelvic CT examination is mandatory in patients with indeterminate renal mass (2, 5).

The histologic classification and grading of renal tumors are also important, as the prognostic and therapeutic implications vary among histologic subtypes. The current 2016 World Health Organization (WHO) classification differentiates between numerous types of kidney tumors including mesenchymal, metanephric, nephroblastic, neuroendocrine, and renal cell tumors among others (3).

Renal cell carcinoma (RCC) is the most common among the neoplastic diseases of the kidney, with approximately 90% of them being diagnosed as RCC (6). RCC is a collective term defining a heterogenous group of neoplasms including 14 subtypes (3) with drastically different histologic appearance, genetics, and prognosis, all originating from the renal tubular epithelium (6). The most common subtypes of RCC are clear cell renal cell carcinoma (ccRCC), papillary cell renal cell carcinoma (pRCC), and chromophobe cell renal cell carcinoma (chRCC), respectively, accounting for about approximately 75, 15, and 5% of all RCC cases (7).

Previous studies proved that the histologic subtype is an independent predictor of patient survival, and patients with ccRCC have a poorer prognosis compared to those with pRCC or chRCCs (8, 9), also patients with ccRCC are most likely to have distant metastasis at the time of radical nephrectomy (10). Due to the markedly higher biological aggressiveness of ccRCC compared to other subtypes, recent practice guidelines divide RCCs into two main groups as ccRCC and non-ccRCC (2, 11).

In the case of advanced RCC, treatment options have been rapidly expanded in the past decades. High-dose bolus interleukin-2 therapy has brought continued good results since approval for the treatment of metastatic RCC in 1992 (12) followed by the era of molecularly targeted therapies and more recently, the era of immunotherapeutic agents (13). Molecularly targeted therapies including Vascular Endothelial Growth Factor (VEGF) targeted tyrosine-kinase inhibitors such as bevacizumab, sunitinib, and pazopanib have been used with great success in patients with metastatic ccRCC, which is currently the recommended first-line standard-of-care treatment according to the European Society for Medical Oncology (ESMO) in patients with good risk (2). Then, novel immunotherapeutic agents revolutionized the treatment of advanced ccRCC (14). The ESMO guidelines recommend combined immune-checkpoint inhibitor antibody therapy (ipilimumab + nivolumab) as first-line treatment in patients with intermediate or poor-risk (2), and since the same results can be achieved using combined immune checkpoint inhibitors with lower toxicity, the usage of cytokine monotherapy diminished (14). Even though there is ample evidence available for the efficiency of sunitinib as a treatment for metastatic ccRCC, other less common renal carcinomas are less researched since they are most often excluded from the controlled phase III trials. Smaller prospective studies, however, suggest that VEGF inhibitors and mammalian target of rapamycin inhibitors are also beneficial in these cases (2). However, pRCCs show a worse response to VEGF-targeted antiangiogenic agents than ccRCCs (15).

Therefore, the non-invasive, imaging-based differentiation between tumor subtypes could facilitate the prediction of patient prognosis and guide clinicians in therapeutic decision-making and follow-up strategies (16). It has been proved that the different subtypes of RCCs have different contrast enhancement dynamics, ccRCCs have peak enhancement on the corticomedullary phase, meanwhile, pRCCs and chRCCs reach the peak during the nephrographic phase (17). Previous studies showed that, relative contrast enhancement of kidney tumors to the renal cortex (18) and CT imaging traits such as heterogeneous contrast enhancement, enhancement degree in corticomedullary phase, the presence of necrosis, and the presence of calcification show association with RCC subtypes (19). However, the morphology-based, conventional radiological evaluation of CT scans is subjective, has low specificity in differentiating RCC subtypes (20), and is highly dependent on the expertise of the radiologists (21).

In 2012, the term radiomics was introduced by Lambin et al. which refers to the automated analysis of medical images by the extraction of an extensive number of quantitative features that can objectively describe the given region of interest (ROI) (22). Radiomics as per definition is the mining and analysis of quantitative features from radiologic images, to improve clinical decision-making by identifying predictive imaging biomarkers and constructing different diagnostic and prognostic models. This novel technique has the potential to detect subtle differences in tissue texture that may not be detected by the human eye (22).

A typical radiomic study comprises the following main steps: medical image acquisition, image pre-processing, segmentation, feature extraction, feature selection, exploratory analysis, and model building and evaluation (23). Conventional radiomics analysis requires lesion segmentation in order to compute hand-crafted radiomics features. The segmentation can be performed either manually by using semi-automatic tools, or fully automatically with the help of convolutional neural networks. In radiomics studies of kidney tumors, the most widely used method is still the manual segmentation (24).

Radiomics analysis allows the extraction of a huge number of quantitative features from the selected volume of interest (VOI) that refer to the intensity histogram, the shape, or the texture of a certain lesion. The definitions and the mathematical formulas of radiomics features can differ between studies, therefore the Imaging Biomarker Standardization Initiative (IBSI) was established as an independent international collaboration aiming to standardize the extraction of quantitative imaging biomarkers to improve the reproducibility of radiomics studies. For a more detailed description of radiomics features, we refer the readers to the Reference Manual of the IBSI updated in 2020 (25). Radiomics analysis is most commonly applied to CT scans given its wide availability. CT texture analysis (CTTA) on contrast-enhanced CT scans also provides a quantitative description of the tissue contrast enhancement distribution after contrast-agent injection.

Radiomics is usually combined with machine learning algorithms for prediction model building. However, the usage of a large number of radiomics features often results in overfitting of the prediction model; therefore the number of features must be effectively reduced before model building (26). As an initial feature-selection step, it is recommended to filter out highly correlated, redundant features (23, 26). The most popular supervised feature selection methods are the model-based wrappers including the so-called recursive feature elimination algorithm that is used to select the optimal subset of predictive features that maximize the prediction performance; and the embedded algorithms such as the least absolute shrinkage and selection operator (LASSO) regression that allows selecting the most predictive features based on the feature importance score (23).

The most widely used conventional machine learning algorithms for prediction model building are logistic regression, LASSO, random forest (RFC), and support vector machine (SVC) classifiers (27).

Previously published studies have focused mainly on distinguishing between benign and malignant renal lesions (28–30) or on identifying aggressive tumor features of ccRCCs (31–37), and only a minority of studies have sought to distinguish between subtypes of RCC (20, 38–41). A few studies also showed that radiomics analysis combined with machine learning could facilitate the non-invasive diagnostics of kidney cancers including both classification of renal tumors, prediction of nuclear grade, identification of patients with poor prognosis, and prediction of treatment response (42, 43). However, most of the previously published studies had a single-center study design and used only internal validation for model evaluation and have not validated their results on external test cases (24, 43).

Yu et al. were among the first who used CT texture analysis for distinguishing between RCC subtypes (41). The authors performed radiomics analysis on 10 selected cross-sectional areas of the tumors in the nephrographic (NG) phase and extracted 43 features. Their SVC trained by all the 43 radiomics features achieved AUCs of 0.91, 0.92, and 0.85 in differentiating between ccRCCs vs. pRCCs, chRCCs and oncocytomas; pRCCs vs. ccRCCs, chRCCs and oncocytomas, and chRCCs vs. pRCCs, ccRCCs, and oncocytomas, respectively. Yu et al. demonstrated the ability of first-order statistics and texture features to predict RCC subtypes (41). By analyzing triphasic CT scans of 143 ccRCCs and 54 non-ccRCCs, Chen et al. illustrated that the radiomics features extracted from the corticomedullary (CM) phase have similar diagnostic ability compared to those extracted from the NG phase in differentiating between ccRCCs and non-ccRCCs (38). In their recent study, Wang et al. analyzed 147 ccRCCs and 43 non-ccRCCs and built a RFC, an SVC, and a logistic regression algorithm-based machine learning model from four selected radiomics features. The models achieved good to excellent results on the internal test dataset with AUC of 0.841–0.909 (20), and the authors also demonstrated that these radiomics-based machine learning models can overperform the diagnostic performance of an expert radiologist (AUC of 0.69). However, in these single-center studies, the machine learning prediction models were not validated on independent external test cases.

External validation of the machine learning models was completed in a two-center study by Li et al., who performed 3D texture analysis on both the unenhanced (UN), CM, and NG phase CT scans of 170 patients (40). In this study, either the Boruta or the minimum redundancy maximum relevance ensemble (mRMRe) algorithms were used to select the most relevant radiomics features. RFC models built in this study were tested on 85 independent external test cases from another hospital. The Boruta-based RFC achieved excellent performance with an AUC of 0.949 while the mRMRe-based RFC achieved an AUC of 0.851. The two sets of selected radiomics features differed significantly, suggesting that there is a huge difference in the performance of the feature selection algorithms, which significantly affects the performance of the machine learning classifier. These results also indicate that the CM features have higher diagnostic ability compared to NG phase features in the differentiation of ccRCCs from non-ccRCCs (40).

Kocak et al. were among the first, who validated their machine learning models’ performance on publicly available datasets (39). In their retrospective study, the authors collected 48 ccRCCs, 13 pRCCs, and 7 chRCCs and performed CT texture analysis on UN and CM phase CT scans to differentiate between RCC subtypes. For external validation 26 cases (13 ccRCCs, 7 pRCCs, and 6 chRCCs) were selected from the TCGA public datasets including The Cancer Genome Atlas-Kidney Renal Clear Cell Carcinoma (TCGA-KIRC) (44, 45), the TCGA-Kidney Renal Papillary Cell Carcinoma (TCGA-KIRP) (44, 46), and the TCGA-Kidney Chromophobe (TCGA-KICH) (44, 47). The authors performed radiomics analysis on the largest cross-sectional areas of the tumors by extracting 275 radiomics features from both the UN and the CM phases. After feature selection, artificial neural network (ANN)-based and SVC-based prediction models were constructed for the differentiation between ccRCC and non-ccRCCs. The ANN algorithm-based model trained on CM phase features achieved an AUC of 0.822, while the SVC reached an AUC of 0.793 on the external test set (39).

Our study aimed to construct a 3D CTTA-based machine learning model for differentiating ccRCC from non-ccRCC that is generalizable and robust against different institutional imaging protocols. We aimed to demonstrate that our radiomics-based machine learning model can achieve comparable results with an expert radiologist. And the final aim of this study was to validate our prediction models on external test cases of a publicly available dataset to prove the models’ reliability.

## Materials and methods

### Patient population

The institutional ethics committee of our university has approved the present study based on the World Medical Association guidelines and the Declaration of Helsinki, revised in 2000 in Edinburgh. As this is a retrospective study, the need for written patient consent was waived by the ethics committee. All patient data were analyzed anonymously.

Preoperative contrast-enhanced abdominal CT scans were retrospectively collected from patients who had undergone either radical or partial nephrectomy between 2008 January and May 2021 at our institution. Out of the patients who had undergone nephrectomy, 551 had available preoperative CT scans. The preoperative unenhanced UN, CM, and excretory (EX) phase CT scans in this study were obtained from the picture archiving and communication system (PACS) of our hospital. 346 cases were excluded due to the following exclusion criteria: diagnosed with benign kidney tumor (*n* = 33), diagnosed with other types of malignant kidney tumor (*n* = 107), nephrectomy due to other reason than tumor (*n* = 75), no available histopathologic report (*n* = 30), dual-phase (UN, CM, and EX) CT scan was not available (*n* = 61), underwent radiofrequency ablation (*n* = 2), damaged DICOM file (*n* = 44), incomplete coverage of the tumor (*n* = 1).

The final patient cohort included 209 patients diagnosed with either ccRCC, pRCC, or chRCC. The final histopathological diagnosis of RCC subtypes served as the reference standard. After nephrectomy, the whole tumor specimens were transferred to histological processing. The official pathology reports were retrospectively collected from the hospital information system. Three patients had two histologically proven tumors, therefore, the final dataset consisted of 161 ccRCCs, 34 pRCCs, and 17 chRCCs.

### Imaging protocols

We examined the patients according to our routine diagnostic protocols with either a 16-slice Brilliance or a 64-slice Ingenuity Core 64 CT scanner (Philips Healthcare, Best, the Netherlands). The following acquisition parameters were used: tube voltage of 100–140 keV; automatic tube current modulation in the range of 105–977 mAs in CM, 93–918 mAs in UN, and 80–910 mAs in EX phase; collimation of 16 mm × 1.5 mm or 64 mm × 0.625 mm for the 16 and 64-slice scans, respectively. The 16-slice acquisitions were routinely reconstructed with filtered back projection (FBP) and 64-slice scans with the iDose4™ hybrid iterative reconstruction kernel. The reconstructed slice thickness was 1.25–5 mm. A non-ionic, iodinated contrast agent (range of concentration: 350–370 mg/ml) was administered intravenously using a power injector with an injection rate of 1.5–3.5 ml/s, while the amount of the injected contrast media was adjusted to the body weight (0.5 g iodine/kg). After contrast agent administration, the CM phase was scanned at 30–45 s, and the EX phase at 300–480 s.

### External test set

For the external validation of our machine learning prediction model, we included cases from the 2019 Kidney and Kidney Tumor Segmentation Challenge (KiTS19) public database (48, 49) that had available dual-phase (UN, CM, and EX phase) CT scans. We identified 75 cases with dual-phase CT scans, from those 69 cases were diagnosed with either ccRCC, pRCC, or chRCC. One case was excluded because the patient’s position on the EX phase scan was prone instead of supine. The CT scans were performed by a variety of scanners including 19 different models from four vendors. The slice thickness varied between 1 and 7 mm, the tube voltage was between 100 and 140 keV, and the tube current varied between 95 and 747 mAs in the CM, 80–667 mAs in the UN, and 80–664 mAs in the EX phase scans. One patient had three tumors, and three had two tumors, therefore the final external test set consisted of 73 lesions. According to the available metadata, 50 of those were ccRCCs, 13 were pRCCs, and 10 were chRCCs. In the KiTS19 dataset, the binary segmentation masks were also available to all the tumors. The results shown here are in whole or part based upon data from the C4KC-KiTS dataset of The Cancer Imaging Archive (TCIA) (44, 48, 49).

### Subjective classification

For the subjective, imaging feature-based analysis, an expert radiologist with over 10 years of experience in urologic imaging classified all the lesions of both internal and external test sets according to the RCC subtypes blinded to the patients’ history, medical records, and to the results of tumor segmentation.

### Image processing and radiomics analysis

Preoperative axial CT scans were anonymized and exported from the institutional PACS in Digital Imaging and Communications in Medicine (DICOM) format. The DICOM files were then converted to NIfTI file format for further image processing and analysis. The image processing and segmentation steps were completed by using the 3D Slicer software v.4.10.2 (50).

**FIGURE 1:**
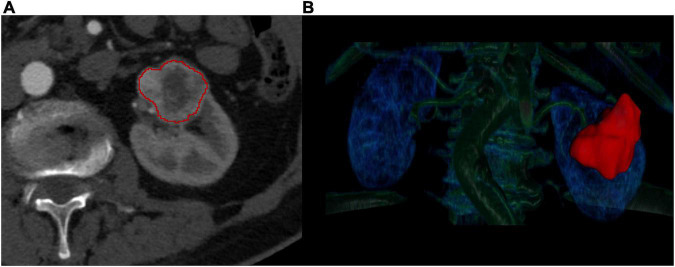
Manual segmentation of kidney tumors. The manual segmentation of the kidney tumors was completed on the corticomedullary phase axial CT scans **(A)**. The entire lesion volume was delineated slice-by-slice **(B)** in order to perform a three-dimensional radiomics analysis.

**FIGURE 2:**
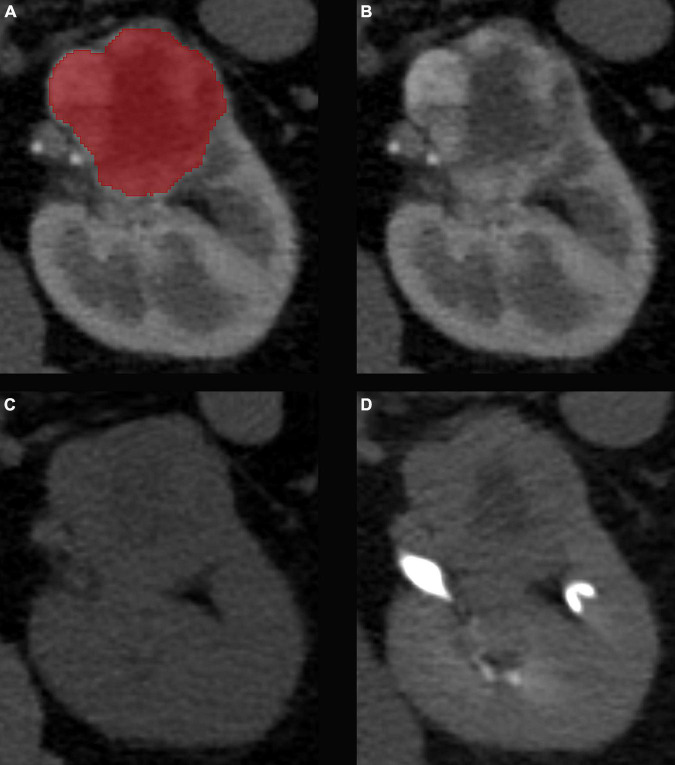
Results of the image co-registration. The manual segmentation of the kidney tumors was completed on the corticomedullary phase CT scans **(A)**. A non-rigid image co-registration was performed to fit the unenhanced **(B)** and excretory **(C)** phase CT scans to the corticomedullary phase as reference **(D)**.

The entire volume of the tumors was segmented slice-by-slice on the CM phase scans. The segmentation of kidney tumors was performed by a trainee with 4 years of experience in tumor segmentation under the supervision of an expert radiologist with over 15 years of experience in abdominal and urologic imaging (Figure 1). The segmentation was performed by avoiding the edge of the tumor to avoid the inclusion of peripheral fat and partial volume effect. The UN and EX phase CT scans were coregistered to the CM phase scans by using the Elastix extension of 3D Slicer (Figure 2).

To minimize the individual patient factors, the inter-scanner differences, and the difference between institutional imaging protocols, the voxel density values of the CM phase CT scans were normalized to the cortical density. In each case, the density of the renal cortex was measured by using 3–3 circular region of interests (ROI), then the mean cortical density was obtained by calculating the average value of the three measurements. In each case, the mean cortical density value was subtracted from the individual voxel intensity values.

For the radiomics analysis, the images were resampled to an isotropic voxel size of 1 mm × 1 mm × 1 mm to get rotation invariant radiomics features and to improve the robustness and reproducibility of the extracted features. The radiomics analysis was performed with the pyRadiomics package (51). A fixed bin width of 16 was used during the calculation of texture features. Altogether, 107 radiomics features were calculated from each phase scan, including 18 first-order histogram-based statistical features, 14 shape-based features, 24 gray-level co-occurrence matrix-based features (GLCM), 16 gray-level run-length matrix-based features (GLRLM), 16 gray-level size zone matrix-based features (GLSZM), 14 gray-level dependence matrix-based features (GLDM), and 5 neighboring gray-tone difference matrix-based features (NGTDM). Data is available in Supplementary Table 1.

### Feature selection

Our feature selection method included three steps, all of which were completed by using solely the training set. First, highly correlated features were filtered out based on Pearson’s correlation coefficients (*r* > 0.95). Then, reproducibility analysis was performed by using intraclass correlation coefficient (ICC) analysis. For the reproducibility analysis, the area of the segmented tumor masks was eroded by 1–1 voxel in each direction as proposed previously (52, 53), and the radiomics feature extraction was repeated. The ICC was calculated for each radiomics feature based on a 2-way, single-rater, absolute agreement model. Only the features with excellent reproducibility defined as ICC value ≥0.90 were included in the wrapper-based feature selection step. The final step included either a least absolute shrinkage and selection operator (LASSO) algorithm, or a tuned ReliefF (TuRF) algorithm which selected the most relevant features based on their feature importance score. The optimal hyperparameter (λ) for LASSO feature selection was automatically determined on the training dataset by using the grid search method with 5-times repeated 5-fold stratified cross-validation. During hyperparameter tuning, negative mean squared error was used as a performance metric that the grid search tried to maximize.

### Machine learning – Model building

**FIGURE 3:**
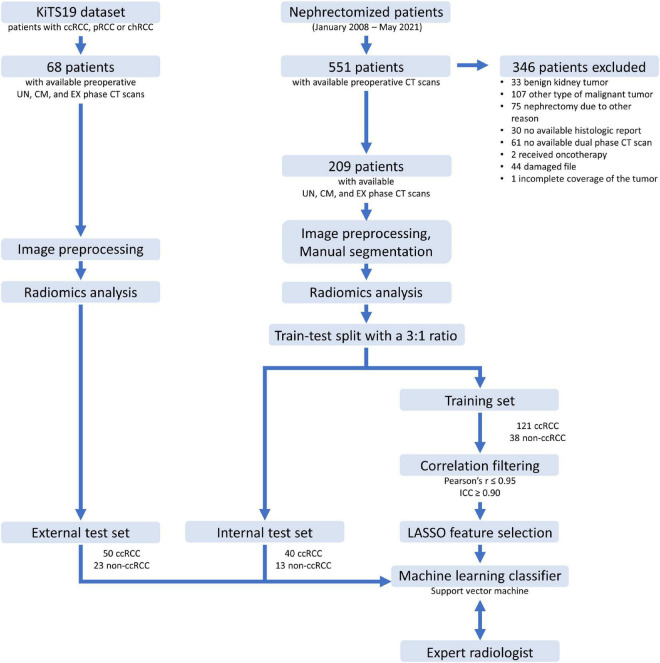
Flowchart of the data analysis steps. ccRCC, clear cell renal cell carcinoma; pRCC, papillary cell renal cell carcinoma; chRCC, chromophobe renal cell carcinoma; KiTS, kidney tumor segmentation dataset; UN, unenhanced; CM, corticomedullary; EX, excretory; LASSO, least absolute shrinkage and selection operator; ICC, intraclass correlation coefficient.

For the machine learning-based analysis, the cases were randomly split into training and test sets with a 3:1 ratio. The radiomics features of the training dataset were standardized by centering around the mean with a unit standard deviation (SD). The test dataset was transformed using the hyperparameters from the training dataset. From the features selected by LASSO, SVC-based machine learning models were constructed to differentiate ccRCCs from non-ccRCCs. From the radiomics features selected by the TuRF algorithm, random forest classifier-based models were constructed. The hyperparameters of the classifiers were optimized with the grid search method based on the accuracy score during five-times repeated 5-fold stratified cross-validation on the training set. To overcome the class imbalance issue, balanced class-weights were used while fitting the models. The diagnostic performance of the models was evaluated on both the training set, the internal test set, and the external test set based on the receiver operating characteristic curve (ROC) analysis. During ROC analysis, the ccRCC data were set as the positive class, while the non-ccRCC as the negative class. The sensitivity, specificity, positive predictive value (PPV), negative predictive value (NPV), and area under curve (AUC) values were calculated. A two-tailed *p*-value < 0.05 indicated statistical significance. Figure 3 shows the main steps of the data analysis.

### Statistical analysis

The continuous variables in ccRCC and non-ccRCC patient groups were checked for homogeneity of variance with the F-test and normal distribution with the Shapiro-Wilk’s test. Categorical variables were compared between the two groups with the chi-squared test and continuous variables with the Mann-Whitney *U*-test. The 95% confidence interval (CI) of the AUC values were calculated based on DeLong’s method. The best threshold was determined based on the “closest top left” method; the point on the ROC curve closest to the top left corner of the plot was defined as min[(1-sensitivities)^2^ + (1-specificities)^2^]. The statistical comparisons between the ROC curves were performed according to the DeLong test. The threshold of *p* < 0.05 was applied to determine significance in all comparisons.

The statistical analysis was completed with “sklearn,” “skrebate,” “statmodels,” and “scipy” packages written in Python (v.3.7.11.) computer language, and with “dplyr,” “stats,” “pROC,” and “irr” packages written in R (v.3.6.3.) computer language.

## Results

### Patient population

The final study population contained 209 patients with 212 tumors (161 ccRCCs and 51 non-ccRCCs). There were no differences in patient age (*p* = 0.079) or sex (*p* = 0.9782) comparing ccRCCs with non-ccRCCs (Table 1). For the machine learning-based analysis, the cases were randomly split into training and test sets with a 3:1 ratio. The distribution of RCC subtypes in the training dataset was ccRCC in 121 cases and non-ccRCC in 38 cases (25 pRCC and 13 chRCC), meanwhile the internal independent test set contained 40 ccRCCs and 13 non-ccRCCs (9 pRCC and 4 chRCC).

**TABLE 1:** Distribution of demographics and tumor types in the patient cohorts.

	Study population			External test set		
		
	ccRCC	non-ccRCC	*P*-value	ccRCC	non-ccRCC	*P*-value
Number of cases (*n*)	161	51	–	50	23	–
Male, *n* (%)	107 (66.5%)	34 (66.7%)	0.978	33 (66.0%)	10 (43.5%)	0.069
Age, median (IQR) years	64.2 (15.6)	66.8 (16.6)	0.079	60.5 (23.2)	53 (13.0)	0.556

IQR, interquartile range; ccRCC, clear cell renal cell carcinoma.

From the KiTS19 public dataset 68 cases with 73 tumors were included in this study as an external test set. In the ccRCC group 33 (66.0%) patients were male and 17 (34.0%) were female, while in the non-ccRCC group, 10 (43.5%) were male and 13 (56.5%) were female (*p* = 0.069). The median age and interquartile range were 60.5 (23.2) years for ccRCCs and 53 (13.0) years for non-ccRCCs (*p* = 0.556).

### Feature selection

During radiomics analysis, 107 radiomics features were extracted from both CM, EX, and UN phase scans. After filtering out the highly correlated and non-robust features, 39 CM, 38 EX, and 35 UN phase features remained. During hyperparameter tuning of the LASSO algorithm, the grid search defined 0.01 as the optimal λ value. In all three cases, an optimized LASSO algorithm (λ = 0.01) was used to select the most predictive radiomics features based on the feature importance score, which selected 10 CM phase, 5 EX phase, and 9 UN phase features. The selected radiomics features included both shape-based features, first-order statistics, and texture features in each case. The selected features are listed in Table 2.

**TABLE 2:** List of the selected radiomics features.

	Corticomedullary phase	Excretory phase	Unenhanced phase
Shape-based	Flatness; sphericity	Sphericity; SurfaceVolumeRatio	Sphericity; SurfaceVolumeRatio
First-order	10th percentile; energy; mean	Energy; median	Entropy; InterquartileRange;Median
Texture feature	GLCM_Correlation; GLRLM_GrayLevelNon-Uniformity; GLRLM_LongRunEmp; GLDM_DependenceNon-UniformityNorm; NGTDM_Coarseness	GLDM_DependenceEntropy	GLCM_InverseVariance; GLDM_DependenceEntropy; GLSZM_LargeAreaEmphasis; GLSZM_SizeZoneNon-UniformityNormalized

GLCM, gray-level co-occurrence matrix; GLRLM, gray-level run-length matrix; GLSZM, gray-level size zone matrix; GLDM, gray-level dependence matrix; NGTDM, neighboring gray-tone difference matrix.

### Machine learning

**FIGURE 4:**
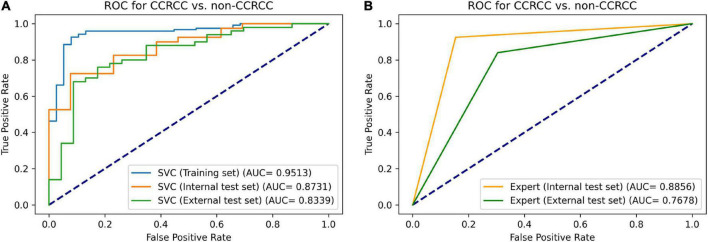
Receiver operating characteristic curves for distinguishing ccRCCs from non-ccRCCs. The performance of our support vector classifier **(A)** was similar to that of a radiologist specializing in urological imaging **(B)**. The radiomics-based machine learning model achieved an AUC of 0.951, 0.873, and 0.834 on the training set, internal test set, and external test set, respectively. Meanwhile, the expert radiologist reached an AUC of 0.886 on the internal test set, and an AUC of 0.768 on the external test set. ROC, receiver operating characteristic curve; SVC, support vector classifier; ccRCC, clear cell renal cell carcinoma.

The optimized SVC model (kernel: rbf, C: 500, gamma: 0.005) trained on the CM phase radiomics features achieved the highest prediction performance in differentiating ccRCCs from non-ccRCCs. During ROC analysis, its performance on the training set was AUC of 0.951 [95% CI: 0.913–0.989], accuracy of 0.925, sensitivity of 0.926, and specificity of 0.921 at threshold 0.655, it also achieved very good prediction rate on the internal independent test set with AUC of 0.873 [95% CI: 0.774–0.973], accuracy of 0.811, sensitivity of 0.90, and specificity of 0.539, and its diagnostic accuracy proved to be robust during validation on external test cases with AUC of 0.834, accuracy of 0.781, sensitivity of 0.800, and specificity of 0.739 (Figure 4). We also compared the diagnostic value of this model against the accuracy of an expert radiologist, which showed comparable results with no significant difference on either the internal (*p* = 0.866) or the external (*p* = 0.256) test sets (Table 3). On the internal test set, the expert radiologist achieved slightly better performance with an AUC of 0.886 (vs. 0.873), accuracy of 0.906 (vs. 0.811), sensitivity of 0.925 (vs. 0.90) and specificity of 0.846 (vs. 0.539), while on the external test set, the SVC slightly overperformed the expert radiologist, who achieved an AUC of 0.768 (vs. 0.834), accuracy of 0.795 (vs. 0.781), sensitivity of 0.84 (vs. 0.80), specificity of 0.696 (vs. 0.739) (Figure 4).

**TABLE 3:** Diagnostic performance of the machine learning models compared to that of an expert radiologist.

	AUC	Threshold	Accuracy	Sensitivity	Specificity	PPV	NPV
SVC - Training set	0.951 [0.913–0.989]	0.655*	0.925	0.926	0.921	0.974	0.796
SVC - Internal test set	0.873 [0.774–0.972]	0.655	0.811	0.900	0.539	0.857	0.636
SVC - External test set	0.834 [0.730–0.938]	0.655	0.781	0.800	0.739	0.870	0.630
RFC - Training set	1.000 [1.000–1.000]	0.500*	1.000	1.000	1.000	1.000	1.000
RFC - Internal test set	0.874 [0.755–0.993]	0.500	0.868	0.950	0.615	0.884	0.800
RFC - External test set	0.663 [0.529–0.796]	0.500	0.685	0.900	0.217	0.714	0.500
Expert – Internal test set	0.886 [0.776–0.996]	0.500	0.906	0.925	0.846	0.949	0.786
Expert – External test set	0.768 [0.659–0.877]	0.500	0.795	0.840	0.696	0.857	0.667

*The optimal threshold was determined based on the point closest to the top left corner of the graph.

AUC, area under the receiver operating characteristic curve; NPV, negative predictive value; PPV, positive predictive value; RFC, random forest classifier; SVC, support vector classifier.

The optimized RFC model (criterion: entropy, n_estimators: 50) trained on the 10 CM phase radiomics features selected by the TuRF algorithm was able to distinguish between ccRCC vs. non-ccRCCs with an AUC of 1.000 on the training set, and also overperformed the SVC model on the internal test set (AUC of 0.874 vs. 0.811), however, showed poor results during external validation with an AUC of 0.663, which indicates overfitting and demonstrates that the LASSO + SVC model can overperform the TuRF + RFC model in this task.

The optimized SVC (kernel: rbf, C: 75, gamma: 0.05) trained by the EX phase radiomics features showed worse performance on both the internal and external test sets with AUC of 0.719 and 0.64, respectively. As expected, the optimized SVC (kernel: linear, C:200) trained on the UN phase features showed even poorer performance with an AUC of 0.725 on the internal test set and AUC of 0.598 on the external test set. The UN and EX phase features were not able to increase the diagnostic performance of the SVC trained on CM phase features, the combined model (kernel: rbf, C: 500, gamma: 0.005) achieved an AUC of 0.862 and 0.711 on the internal and external test sets, respectively.

In Supplementary Table 2, we compare the results of our machine learning models with those reported in previously published studies.

## Discussion

In this study, we constructed an externally validated radiomics-based machine learning prediction model for the differentiation of ccRCC from non-ccRCC. Our SVC algorithm-based machine learning model trained by CM phase features achieved very good performance on the independent test cases from our institute with an AUC of 0.87 and its diagnostic ability also proved to be reproducible with an AUC of 0.83 during validation on external test cases from the KiTS19 dataset. In addition, we evaluated the accuracy of our SVC against that of an expert radiologist, which showed that the performance of the machine learning model is comparable (accuracy of 0.79 vs. 0.78 on the external dataset) which further supports the current literature and demonstrates the potential of CT texture analysis in this application.

The majority of the previously published studies focused on differentiating between benign and malignant kidney lesions (28–30) or identifying aggressive tumor features of ccRCCs (31–37), and only a handful of studies aimed to distinguish between the RCC subtypes (20, 38–41). It is important to highlight that previous studies used different softwares for radiomics feature extraction including both in-house developed algorithms (40, 41), and open-source tools such as the MaZda software (39) and the pyRadiomics package (38) which complicates the direct comparison of the previously published results. More importantly, most of the previous studies had a single-center study design and their models had not been validated on independent, external cases.

Yu et al. were among the first who used CT texture analysis for distinguishing between RCC subtypes (41). The authors performed radiomics analysis on 10 selected cross-sectional areas of the tumors in the NG phase and extracted 43 features. In each case, the average of the 10 values per feature was calculated. A 5-fold cross-validated, linear SVC was built to differentiate RCCs from oncocytomas for each radiomics feature separately. In distinguishing between ccRCCs vs. pRCCs, chRCCs and oncocytomas, first-order statistics “geometric mean” achieved the best predictive value with an AUC of 0.809. In the task of distinguishing between pRCCs vs. ccRCCs, chRCCs and oncocytomas, first-order statistics “median” reached the highest performance with an AUC of 0.811. While in the prediction of chRCCs vs. pRCCs, ccRCCs, and oncocytomas none of the features achieved good diagnostic performance: the highest AUC was 0.757. The SVC trained by all the 43 radiomics features achieved AUCs of 0.91, 0.92, and 0.85 in the three tasks, respectively, which may indicate that the prediction performance of the combination of the radiomics features is superior compared to the diagnostic value of individual features. Yu et al. demonstrated the ability of first-order statistics and texture features to predict RCC subtypes, however, in this single-center study all the scans were performed on the same CT scanner and the results were not validated on an independent test set (41). Yu et al. built SVC models from NG phase radiomics features, while in our study, we built an SVC prediction model from the combination of the most predictive CM features that proved to be reproducible when tested on independent external test cases (41). Our prediction model achieved comparable results compared to those reported by Yu et al. (AUC of 0.87 on the internal test set vs. AUC of 0.91 during cross-validation), which may indicate that the performance of CM features and NG features is comparable in predicting ccRCCs, although Yu et al. also included 10 oncocytomas in their dataset (41).

Chen et al. retrospectively collected triphasic CT scans from patients with RCCs (38). The final cohort in this study included 143 ccRCCs and 54 non-ccRCCs. To extract non-textural features, the authors calculated 13 different absolute and relative enhancement and attenuation ratios and values. After radiomics analysis, LASSO was used to select the most important features and to calculate texture-score with the linear combination of the selected features. Finally, three different prediction models were built, one logistic regression-based model from non-texture features, one model from texture features, and a third, combined logistic regression model. Among both the non-textural and the texture-feature-based models, the CM phase models achieved the highest performance with AUC = 0.823 and 0.887, while the performance of the combined model showed similar results in the CM and NG phases with AUCs of 0.891 and 0.900. The results of this study showed that adding non-texture features can improve the prediction performance of the texture feature-based model and that the CM phase and the NG phase radiomics features have similar diagnostic ability in differentiating between ccRCCs and non-ccRCCs. However, these models were not validated on an independent test set in this manuscript (38). The results of this study are comparable with the results of our SVC model trained on the CM phase radiomics features, especially with the results we reported on the training set (AUC = 0.951), however, we also validated the performance of our model on both independent internal (AUC = 0.873) and external test cases (AUC = 0.834).

In their recent study, Wang et al. analyzed 147 ccRCCs and 43 non-ccRCCs and built a RFC, an SVC, and a logistic regression algorithm-based machine learning model from four selected radiomics features (20). The authors reported very good results on the internal test dataset for each machine learning algorithm. Their RFC achieved the highest diagnostic performance with an AUC of 0.909 followed by the logistic regression classifier with an AUC of 0.906, while the SVC showed slightly worse results with an AUC of 0.841 (20). These results on the independent internal test set (AUC = 0.841–0.909) are very similar to the results of our SVC (AUC of 0.88) on the independent internal test set. However, all patients were scanned with the same CT scanner in this single-center study, and the models were not validated on external test cases. The diagnostic performance of an expert radiologist was also reported in this study, and the authors successfully demonstrated that radiomics-based machine learning models can overperform the accuracy of an expert radiologist. Although, the radiologist’s performance reported in this manuscript was slightly inferior to that of our study (AUC of 0.69 vs. 0.76–0.88, sensitivity of 0.85 vs. 0.84–0.93, and specificity of 0.58 vs. 0.70–0.85).

In a two-center study by Li et al., external validation of the machine learning models was also completed (40). The authors performed 3D texture analysis on both the UN, CM, and NG phase CT scans of 170 patients. After the extraction of 3 × 52 texture features from the tumors, either the Boruta algorithm or the minimum redundancy maximum relevance ensemble (mRMRe) was used to select the most relevant features. Two RFCs were trained, one with the 8 CM phase features selected by the Boruta algorithm, and one by the combination of 7 nephrographic and one CM phase features selected by the mRMRe algorithm. The machine learning models were tested on 85 independent external test cases from another hospital. The Boruta-based model achieved an AUC of 0.949 and an accuracy of 92.9%, which significantly overperformed the mRMRe-based model which reached an AUC of 0.851 and an accuracy of 81.2%. Their results suggest that there is a huge difference between the performance of feature selection algorithms, as the two sets of selected features were markedly different. These results also indicate that the CM features have higher diagnostic ability compared to NG phase features in the differentiation of ccRCCs from non-ccRCCs (40). In our study, we extracted not just second-order texture features, but also first-order statistical parameters and shape-based features from the tumor volumes in the CM phase. The LASSO algorithm selected two shape-based, three first-order, and five texture features as the most important ones, which may indicate the importance of first-order statistics and shape-based features in addition to texture features. Although our results on the external test set are slightly worse than those reported by Li et al. (AUC of 0.834 vs. 0.949), it could be at least partly due to the fact that our independent test sets contained a significant number of atypical cases which is supported by that the accuracy of our SVC model proved to be comparable with that of an expert radiologist (accuracy of 0.78 vs. 0.79) (40).

Kocak et al. were among the first, who validated their machine learning models’ performance on publicly available datasets (39). In their retrospective study, Kocak et al. collected 48 ccRCCs, 13 pRCCs, and 7 chRCCs and performed CT texture analysis on UN and CM phase CT scans to differentiate between RCC subtypes. For external validation, the authors selected 13 ccRCCs, 7 pRCCs, and 6 chRCCs from three publicly available datasets including The Cancer Genome Atlas-Kidney Renal Clear Cell Carcinoma (TCGA-KIRC) (44, 45), the TCGA-Kidney Renal Papillary Cell Carcinoma (TCGA-KIRP) (44, 46), and the TCGA-Kidney Chromophobe (TCGA-KICH) (44, 47). After manual segmentation, the authors performed texture analysis on the largest cross-sectional areas of the tumors by extracting 275 radiomics features from both the UN and the CM phases. After feature selection, the authors constructed artificial neural network (ANN)-based and SVC-based prediction models, that were evaluated based on ROC curve analysis and Matthews correlation coefficient (MCC) values. In the differentiation between ccRCC and non-ccRCCs, the ANN algorithm-based model combined with adaptive boosting trained on CM phase radiomics features, achieved an AUC = 0.870, accuracy of 86.7%, and MCC = 0.686 during internal validation, and AUC = 0.822, accuracy of 84.6%, and MCC = 0.728 on the external test set. Meanwhile, the SVC combined with adaptive boosting achieved an AUC = 0.852, accuracy of 89.7%, and MCC = 0.745 during internal validation, and AUC = 0.793, accuracy of 65.3%, and MCC = 0.426 on the external test set (39). Our results can be compared with those reported by Kocak et al., our SVC achieved slightly better performance both during internal validation (AUC 0.873 vs. 0.852 and external validation (AUC of 0.834 vs. 0.793), however, it is important to note that for external validation, we used 73 tumors from the KiTS19 dataset, while Kocak et al. validated their results on 26 selected cases from the TCGA datasets (39).

We confirmed the results of previous studies that the CM phase radiomics features are superior compared to the EX phase ones (29, 38). Interestingly, contrary to the results of Raman et al. (29), we were unable to prove that the addition of UN and/or EX phase radiomics features increase the predictive performance of the model, however, we did not analyze NG phase scans as these were not available in the KiTS19 dataset.

The limitation of our study is the relatively low number of patients, and that only the three most common RCC subtypes were studied, however, the other subtypes are rare. The distribution of the RCC subtypes was unbalanced, reflecting the unequal distribution in the global population. To handle unbalanced datasets, instead of using synthetic sampling methods, we used class-weight optimization during “training” and then we tested the model on independent cases from different institutions. Since the inclusion criteria in this study were not strict to avoid selection bias, the internal and external test datasets were also slightly unbalanced reflecting real-world conditions. We decided not to use synthetic sampling techniques to balance the groups of test sets, as we wanted our results on test sets to illustrate how the model would work in the daily clinical practice. The distribution of patients by sex in the training and test datasets were also slightly imbalanced, but it is well known that in the general population men are more likely to be affected by kidney cancer than women and that kidney cancer is about twice as common in men as in women. Accordingly, the number of male patients in our own study was slightly higher than the number of female patients in both our own dataset and the external test set. However, the distribution did not reach a significant level, i.e., the imbalance was similar between the ccRCC and non-ccRCC groups. Finally, nephrographic phase CT scans were not included in our study, as those were not available in the KiTS19 dataset.

In conclusion, we successfully built a support vector classifier-based machine learning model from CM phase radiomics features that was able to differentiate between ccRCCs and non-ccRCCs with good accuracy. The performance of our model was validated on both cases from our own institute during internal validation (AUC = 0.87), and cases from the KiTS19 dataset during external validation (AUC = 0.83), which proved our machine learning model’s reliability and generalizability. We also compared the accuracy of the SVC with that of an expert radiologist (accuracy of 0.79 vs. 0.78 on the external dataset), which showed non-inferior results. Therefore, we conclude that radiomics analysis combined with machine learning could facilitate the non-invasive diagnosis of RCCs in clinical practice in an objective and automated way.

## Data availability statement

The original contributions presented in this study are included in the article/Supplementary material, further inquiries can be directed to the corresponding author.

## Ethics statement

The studies involving human participants were reviewed and approved by Semmelweis University Regional and Institutional Committee of Science and Research Ethics. The ethics committee waived the requirement of written informed consent for participation.

## Author contributions

BB: conceptualization, investigation, data curation, formal analysis, methodology, and writing – original draft. RS, AR, BK, ZZ, and LP: data curation and writing – original draft. BF: methodology, data curation, and writing – review and editing. AS and ES: methodology and writing – review and editing. PM-H: resources and writing – review and editing. PK: conceptualization, investigation, supervision, and writing – review and editing. All authors read and approved the final version of the manuscript.
