# Supplementary material for: Radiomics analysis of contrast-enhanced CT scans can distinguish between clear cell and non-clear cell renal cell carcinoma in different imaging protocols
Source: Front Med (Lausanne). 2022 Oct 13;9:974485. doi: 10.3389/fmed.2022.974485 (PMC9606401; doi:10.3389/fmed.2022.974485)
Supplement: Supplementary file 1 [file Table_1.DOCX]

**Supplementary Table 1.** Comparison of results with the state of the arts.

| **Study** | **Design** | **Dataset** | **Segmentation** | **External test set** | **CT contrast phase** | **Radiomics features** | **Feature selection algorithms** | **Machine learning models** | **Comparison with expert radiologist** | **Results** |
| --- | --- | --- | --- | --- | --- | --- | --- | --- | --- | --- |
| **This study** | ccRCC vs. non-ccRCC | 209 patients  (161 ccRCCs,  34 pRCCs, and 17 chRCCs) | 3D tumor volume | Cases from the KiTS19 public dataset  73 tumors  (50 ccRCCs, 13 pRCCs and 10 chRCCs) | UN, CM, EX | Pyradiomics package  107 features (firstorder statistics, shape-based, GLCM, GLRLM, GLSZM, GLDM, NGTDM) | Pearson’s correlation (r>0.95), ICC (≥0.90), LASSO | SVC | Yes | CM phase SVC model achieved the best performance.  **SVC - CM phase:** AUC=0.87 (internal); AUC=0.83 (external)  **Radiologist:** AUC=0.89 (internal), AUC=0.77 (external) |
| **Yu et al. (41)** | ccRCCs vs. pRCCs, chRCCs and oncocytomas;  pRCCs vs. ccRCCs, chRCCs and oncocytomas;  chRCCs vs. pRCCs, ccRCCs, and oncocytomas;  oncocytomas vs. ccRCC, pRCC, and chRCC | 119 patients (46 ccRCC,  41 pRCC,  22 chRCC, and  10 oncocytomas) | 10 selected cross-sectional slices | No | NG | In-house developed program  43 features (histogram-based, GLCM, GLRLM, GLGM features, and 9 Laws’ features) | No | SVC | No | **SVC – 43 features:** AUCs of 0.91, 0.92, 0.85, and 0.86 |
| **Chen et al. (38)** | ccRCC vs. non-ccRCC | 143 ccRCCs,  25 pRCCs, and 29 chRCCs | 3D tumor volume | No | UN, CM, NG, EX | Attenuation values as ratios as non-textural features,  pyRadiomics package  texture features (GLCM, GLRLM, GLSZM, GLDM, NGTDM features) | T-test, Mann-Whitney U-test, LASSO | LR | No | CM phase models reached the highest AUC values.  **CM – non-textural:** AUC=0.823  **CM – texture-feature:** AUC=0.887  **CM – Combined model:** AUC=0.891  **NG – Combined model:** AUC=0.900 |
| **Wang et al. (20)** | ccRCC vs. non-ccRCC | 190 patients (147 ccRCCs,  24 pRCC,  13 chRCC,  6 collecting duct carcinoma) | 3D tumor volume | No | CM | Artificial Intelligence Kit software  396 features (histogram-based, Haralick, formfactor, GLSZM, GLCM, GLRLM features) | ICC (>0.75), ANOVA test, general linear model, first 10% mutual information, Logistic regression, Correlation | RFC, SVC, LR | Yes | **RFC:** AUC of 0.909;  **LR:** AUC of 0.906;  **SVC:** AUC of 0.841;  **Radiologist:** AUC of 0.69 |
| **Li et al. (40)** | ccRCC vs. non-ccRCC | 170 patients  (120 ccRCCs,  25 pRCCs, and 20 chRCCs) | 3D tumor volume | Cases from external hospital  85 patients  (63 ccRCCs, 11 pRCCs, and  11 chRCCs) | UN, CM, NG | In-house developed program  52 features (GLCM, GLRLM, GLSZM, NGTDM) | ICC (≥0.85), Boruta or mRMRe | RFC | No | **Boruta – RFC:** AUC of 0.949  **mRMRe – RFC:** AUC of 0.851 |
| **Kocak et al. (39)** | ccRCC vs. non-ccRCC | 68 patients  (48 ccRCCs,  13 pRCCs, and 7 chRCCs) | the largest cross-sectional areas of the tumors | Cases from the TCGA public datasets  26 tumors  (13 ccRCCs,  7 pRCCs, and 6 chRCCs) | UN,  CM | MaZda software  275 features (histogram features, gradient features, GLCM, GLRLM, autoregressive model features, and Haar wavelet features) | ICC (>0.80), wrapper-based feature selection | ANN, SVC | No | **ANN – CM:** AUC of 0.870 (internal), AUC of 0.822 (external)  **SVC – CM:** AUC of 0.852 (internal), AUC of 0.793 (external) |

ANN: artificial neural network; ccRCC: clear cell renal cell carcinoma; chRCC: chromophobe cell renal cell carcinoma; CM: corticomedullary phase; CT: computed tomography; EX: excretory phase; GLCM: gray-level co-occurrence matrix; GLDM: gray-level dependence matrix; GLGM: gray-level gradient matrix; GLRLM: gray-level run-length matrics; GLSZM: gray-level size-zone matrix; LR: Logistic regression; mRMRe: minimum redundancy maximum relevance ensemble; NG: nephrographic phase; NGTDM: neighborhood gray tone difference matrix; pRCC: papillary cell renal cell carcinoma; RFC: random forest classifier SVC: support vector classifier; TCGA: The Cancer Genome Atlas; UN: unenhanced phase;
